# Cross-species EST alignments reveal novel and conserved alternative splicing events in legumes

**DOI:** 10.1186/1471-2229-8-17

**Published:** 2008-02-19

**Authors:** Bing-Bing Wang, Mike O'Toole, Volker Brendel, Nevin D Young

**Affiliations:** 1 Department of Plant Pathology, University of Minnesota, St. Paul, MN 55108, USA; 2 Department of Genetics, Development and Cell Biology and Department of Statistics, Iowa State University, Ames, IA 50011, USA; 3 Pioneer Hi-Bred International, Inc., a DuPont company, 7200 N.W. 62nd Avenue, Johnston, IA 50131, USA

## Abstract

**Background:**

Although originally thought to be less frequent in plants than in animals, alternative splicing (AS) is now known to be widespread in plants. Here we report the characteristics of AS in legumes, one of the largest and most important plant families, based on EST alignments to the genome sequences of *Medicago truncatula *(*Mt*) and *Lotus japonicus *(*Lj*).

**Results:**

Based on cognate EST alignments alone, the observed frequency of alternatively spliced genes is lower in *Mt *(~10%, 1,107 genes) and *Lj *(~3%, 92 genes) than in *Arabidopsis *and rice (both around 20%). However, AS frequencies are comparable in all four species if EST levels are normalized. Intron retention is the most common form of AS in all four plant species (~50%), with slightly lower frequency in legumes compared to *Arabidopsis *and rice. This differs notably from vertebrates, where exon skipping is most common. To uncover additional AS events, we aligned ESTs from other legume species against the *Mt *genome sequence. In this way, 248 additional *Mt *genes were predicted to be alternatively spliced. We also identified 22 AS events completely conserved in two or more plant species.

**Conclusion:**

This study extends the range of plant taxa shown to have high levels of AS, confirms the importance of intron retention in plants, and demonstrates the utility of using ESTs from related species in order to identify novel and conserved AS events. The results also indicate that the frequency of AS in plants is comparable to that observed in mammals. Finally, our results highlight the importance of normalizing EST levels when estimating the frequency of alternative splicing.

## Background

Alternative splicing (AS) is an important cellular process that leads to multiple mRNA isoforms from a single pre-mRNA in eukaryotic organisms. Plant AS events used to be regarded as rare. However, a growing number of computational studies have now demonstrated that the frequency of alternatively spliced genes in plants is higher than previously estimated [1,2]. 20–30% of expressed genes are alternatively spliced in *Arabidopsis thaliana *(*At*) and rice (*Oryza sativa, Os*) as revealed by large scale EST-genome alignments [1,2]. A recent study using EST pairs gapped alignments (EST-EST) surveyed 11 plant species and suggested that overall AS frequencies vary greatly in different plant species, with some rates comparable to those observed in animals [3]. In mammals, exon skipping (ExonS) is the most common type of AS [4,5], but in *At *and *Os*, intron retention (IntronR) is most abundant [1]. Alternative acceptor site (AltA) and alternative donor site (AltD) are also common in these two model plants [1,2]. A rare type of AS event is alternative position (AltP), where an alternative intron differs from its constitutive form in both donor and acceptor sites [1]. Examples of all five types of AS events are shown in Additional file 1 (Supplementary Figure S1). Recently, a novel approach involving whole-genome microarray data revealed that IntronR can be detected in ~8% of *At *genes [6]. The prevalent IntronR events suggest that an intron recognition mechanism is predominant in *At *and *Os *[1]. A small fraction of conserved AS events have also been discovered and confirmed between *At *and *Os*, strongly indicating the functional importance of AS in plants [1].

Most computational studies on AS in mammals and plants use transcript sequences from the same species as their genome sequences. For species with relatively small EST/cDNA collections, transcript sequences from closely related species can be a valuable resource for identification of additional AS events. Even for species with large EST collections, including human and mouse, cross-species EST alignment have been used to reveal novel AS events. As many as 42% of human genes show novel AS patterns by aligning mouse transcripts to human genome [7], and more than 10% of human loci exhibit conserved AS events in mouse [8]. Another study applying the cross-species strategy to human, mouse and rat identified 758 novel cassette-on exons (ExonS) as well as 167 novel retained introns (IntronR). RT-PCR validated 50~80% of tested events, indicating the impressive potential of the cross-species method in identifying novel AS events [9]. In plants, cross-species transcripts have been used mainly for gene annotation. For example, transcript assemblies from 185 species were mapped to the *Os *genome, confirming about 90% of gene predictions plus about 500 novel genes [10]. Similarly, approximately 850 novel genes and 1,000 novel AS events were annotated in *Os *by aligning ESTs from seven plant species [11]. The AS events supported by cross-species transcripts are likely to be functional, as they are conserved between species.

Experimental studies provide additional insight into the function of AS in plants. A wide range of plant genes with diverse functions are regulated through AS, including (but not limited to) genes involved in transcription, splicing, photosynthesis, disease resistance, stress, flowering and grain quality (reviewed in [12,13]). Genes involved in splicing, especially in splicing regulation, seem to have a higher frequency of AS [14]. Several recent studies have revealed that serine/arginine-rich (SR) protein transcripts exhibit extensive levels of AS and that some AS pattern are conserved between *At *and *Os *[15-18]. Maize SR protein transcripts are also alternatively spliced [19,20]. Temperature stress (cold and heat) as well as hormone treatment can change the AS patterns of SR proteins in *At*, suggesting an important role for AS in the stress response [15]. One *At *U2AF35 homolog (atU2AF35a) is alternatively spliced by removing non-canonical introns with repeated borders in the 3'-end of the coding region. Changing the expression of U2AF35 homologs alters the splicing pattern of the FCA gene and, in turn, causes variation in flowering time [21]. The U1-70K gene encodes a core protein in U1 small nuclear ribonucleoproteins (snRNP). The sixth intron of U1-70K can be retained in *At *[22], an event conserved between *At *and *Os *[1]. Recently, the IntronR event was experimentally confirmed in *Os *and maize [23].

Over 400 genes in 54 plant species are now known to be alternatively spliced [24]. Only a few AS events, however, have been reported in legumes (*Fabaceae*), one of the largest and most important plant families. In *Lotus japonicus *(*Lj*), a phytochelatin synthase gene (LjPCS2) can be alternatively spliced, with one isoform present in nodules (LjPCS2-7N) and another isoform in roots (LjPCS2-7R). The two isoforms encode proteins differing only in five amino acids, where one protein (LjPCS2-7N) confers cadmium (Cd) tolerance while the other does not, at least not when ectopically expressed in yeast cells [25]. A nodule specific gene (LjNOD70) shows an IntronR event in *Lj*, where the spliced isoform is less abundant in nodules [26]. Six sucrose synthase genes exist in *At*, *Os *and *Lj*, but only the *Lj *homolog (LjSUS2) is alternatively spliced [27]. In soybean (*Glycine max*,*Gm*), a nodule specific gene (GmPGN) has been identified through EST data mining. Experiments confirmed the tissue specificity and also revealed AS events for this gene [28]. In kidney bean (*Phaseolus vulgaris*), a single gene (PvSBE2) can be alternatively spliced to produce two starch-branching enzyme isoforms, each with distinct characteristics and subcellular localization [29]. A highly abundant novel giant retroelement (*Orge*) of pea (*Pisum sativum*) is partially spliced, probably regulating the ratio of full-length protein, as the retained intron causes truncation [30].

Two legume plants, *Medicago truncatula *(*Mt*) and *L. japonicus *(*Lj*), have large-scale genome sequencing projects in progress [31]. In late 2006, the *Medicago *genome sequence consortium (MGSC) constructed a partial genome assembly based on 1,996 Bacterial Artificial Chromosome (BAC) clone sequences as a basis for constructing draft pseudochromosomes. A total of 42,358 genes were annotated by the International *Medicago *Genome Annotation Group (IMGAG) [32], representing ~60% of all *Mt *genes. The data has been released as Mt1.0, available at [33]. In parallel, *Lj *has 1,394 Transformation-competent Artificial Chromosomes (TACs) in GenBank (as of mid-2006), with 488 of them at phase 3 (finished). Both legume model plants have relatively large EST collections (over 150,000 sequences). There are also large numbers of transcript sequences from other legume species, especially soybean. These features make *Mt *and *Lj *ideal for computational comparison of AS events in legume and other plants.

In this study, all available transcript sequences from legumes were aligned to *Mt *and *Lj *BAC/TAC sequences. *At *and *Os *transcript sequences were also aligned to their own genome sequences for comparison purpose. The frequency of alternatively spliced genes is very similar across the different plant species as long as the number of ESTs used as a basis for analysis is standardized across different species. In the case of *Mt*, about 10% of expressed genes are alternatively spliced at current EST coverage, with IntronR the most abundant type. Novel and conserved AS events can be identified if cross-species ESTs are aligned to the genome. These results provide a basis for analyzing AS events conserved in all plants as well as those found in legumes only. This is the first large-scale analysis of AS using EST-genome alignments in plants other than *At *and *Os*, and it is also the first detailed comparison using cross-species transcript sequences in plants.

## Results

### Characteristics of legume exons and introns

Two computer programs, GeneSeqer [34] and GMAP [35], produced largely similar results for the alignment of EST sequences to their native genomes for the *Mt*, *Lj*, *At*, and *Os *data sets. To reduce the likelihood of alignment artifacts as a result of ambiguities, only the commonly predicted alignments from the two programs were used in further analyses. Moreover, highly stringent criteria (>95% sequence identity, >80% transcript coverage) were used to limit the possibility of transcript mapping to non-cognate, diverged locations in the incompletely sequenced genomes. Approximately one half and one third of the species-specific EST sets could be aligned to the current *Mt *and *Lj *genome sequences, respectively, roughly reflecting the coverage of the whole genomes by their current sequence assemblies. For *Lj*, ~15% of the transcript sequences were mapped to finished (phase 3) BAC/TACs. Unless stated otherwise, our analyses for *Lj *were based solely on this subset. As shown in Table 1, a total of 11,516 and 3,298 genes/transcription units (TU, as defined in METHODS) were identified in *Mt *and *Lj*, respectively, with 74% and 57% of them having multiple EST support. The average number of ESTs per gene/TU was 10 and 7 in *Mt *and *Lj*, respectively, compared with 26 and 30 in *At *and *Os*.

**Table 1:** Transcript alignments, intron and exon features in plants

	Medicago	Lotus^#^	Arabidopsis	Rice
EST/cDNA total	225,920	150,855	691,516	1,009,754
Mapped to genome^	104,382 (46.2%)	22,144 (14.7%)*	589,254 (85.2%)	916,825 (90.8%)
Transcription unit (TU)/Genes	11,516	3,298	22,518	31,044
MultiEST TU/Genes	8,544 (74.2%)	1,879 (57.0%)	19,857 (88.2%)	26,859 (86.5%)
Average (Median) ESTs/gene	9.8 (4)	6.9 (2)	26.3 (11)	30.1 (10)
Number of Introns	32,860	4,357	97,095	107,162
Average (Median) intron size	472 (218)	458 (215)	171 (101)	438 (164)
Long intron (>1000 nt)	12.7%	10.9%	0.7%	10.7%
Number of internal exons	24,600	2,717	78,911	83,668
Average (Median) internal exon	140 (108)	127 (100)	164 (114)	175 (113)

^ Transcript sequences are required to have >95% identity and >80% coverage to be considered as mapped.

^# ^Lotus data are based on the ESTs aligned to finished TACs (phase 3).

* A total of 48,691 (32.3% of 150,855) transcript sequences can be mapped to *Lj *TACs in all phases, including phase 1, phase 2 and phase 3.

**Figure 1:**
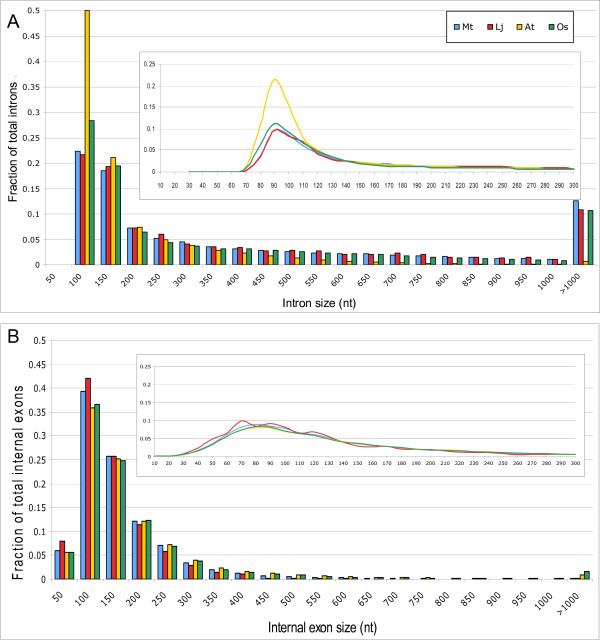
**Size distributions of introns and internal exons in plants**. The x-axis indicates the size of either introns (A) or internal exons (B). Each number except the last one is labeled with the upper bound (e.g., 100 nt comprises size 51–100 nt). The y-axis indicates the fraction of total introns (A) or internal exons (B) for a given size range of intron or internal exon. The insets show a detailed distribution of smaller (<300 nt) introns (A) or internal exons (B). The bin size is 10, and 100 nt comprises size 91–100 nt for the insets.

We compared intron/exon features revealed by EST alignments in the four species. The intron size distribution was quite similar in *Mt *and *Lj*, with a mean intron size around 460–470 nt and median approximately 220 nt in both species. Legume introns are therefore significantly longer than in *At *(mean 171 nt, median 101 nt) and slightly longer than *Os *introns (mean 438 nt, median 164 nt). As shown in Figure 1A, the intron size distributions have a peak near 90 nt in all four species. *Mt *and *Lj *have fewer introns shorter than 150 nt but more introns longer than 200 nt compared with *At *and *Os*. *At *introns are clearly the shortest of the four plants. Fewer than 1% of introns are longer than 1,000 nt in *At*, while this number is over 10% in the other plant species. Exon size tends to be similar among the four plant species, with legume exons slightly shorter than *At *and *Os *exons. In *Mt *and *Lj*, the mean internal exon sizes are 140 and 127 nt, respectively, with the median sizes about 108 nt and 100 nt. *At *and *Os *have internal exons with a mean of 164 nt and 175 nt and a median of 113 nt and 114 nt. Figure 1B shows that the size distributions of exons in *Mt*, *At *and *Os *all display a peak at around 80 nt. *Lj *data is less consistent due to its small sample size. In contrast to introns, the frequency of exons smaller than 150 nt is higher in *Mt *and *Lj *than in *At *and *Os*, while the frequency of exons longer than 200 nt is lower in legumes. Overall, legumes have longer introns but slightly shorter exons than *At *and *Os*. Generally speaking, plant introns are longer than exons. More than 40% of introns in *Mt*, *Lj *and *Os *are longer than 300 nt, while less than 10% exons are so large.

As noted previously [1,36], the GC-content of introns and exons is ~5% lower in *At *than in *Os*. The GC-content of legume introns and exons is very similar to that of *At*, although *Mt *has slightly lower GC-content than either *At *or *Lj *in both intronic and exonic regions (see Additional file 1, Supplementary Table S1 and Supplementary Figure S2). G-content and A-content are similar in all species including *Os*, although *Os *introns are relatively more C-rich and less U-rich. There is more variation in the distribution of U-(T-) and A- content than in G- or C-content in all species (see Additional file 1, Supplementary Figure S3). The difference in GC-content between introns and exons is about 10% in all four species, with *Mt *showing the largest difference of 11.7% and *Os *showing the smallest, 9.6% (see Additional file 1, Supplementary Table S1).

### Different plant species have similar levels of alternatively spliced genes

Previous studies revealed that approximately 20% of expressed genes are alternatively spliced in *At *and *Os*, with half of the AS events being intron retention (IntronR) [1]. When we re-examined AS frequency in *At *and *Os *for this study, we also found a frequency of around 20%. However the total number of transcript sequences increased 80%-200% due to the increased sizes of the EST data sets in these species. In the case of *Mt *and *Lj*, the number of ESTs available for analysis were much lower. Consistently, the fraction alternatively spliced genes observed was much lower, just 9.6% in *Mt *and 2.8% in *Lj *(Table 2). Examples of alternatively spliced genes in *Mt *are shown in Additional file 1, Supplementary Figure S1. All the AS data are deposited and viewable at the ASIP site [37].

**Table 2:** Comparison of alternative splicing events and frequencies in plants

	Medicago	Lotus^#^	Arabidopsis	Rice
AltD	204 (13.5%)	18 (15.7%)	818 (11.3%)	1,165 (9.6%)
AltA	350 (23.1%)	37 (32.2%)	1,785 (24.7%)	2,377 (19.5%)
AltP	21 (1.4%)	2 (1.7%)	106 (1.5%)	306 (2.5%)
ExonS	162 (10.7%)	10 (8.7%)	445 (6.2%)	1,332 (10.9%)
IntronR	778 (51.3%)	48 (41.7%)	4,062 (56.3%)	7,011 (57.5%)

Total	1,515	115	7,216	12,191
AS genes	1,107 (9.6%)	92 (2.8%)	4,497 (20.0%)	6,313 (20.3%)

Percentages in parenthesis for each alternative splicing type are the portion relative to the total events. Percentages for AS genes are the portion of alternatively spliced genes relative to the total number of expressed genes (genes/TU) in Table 1.

^# ^Lotus data are based on the ESTs aligned to finished TACs (phase 3).

**Figure 2:**
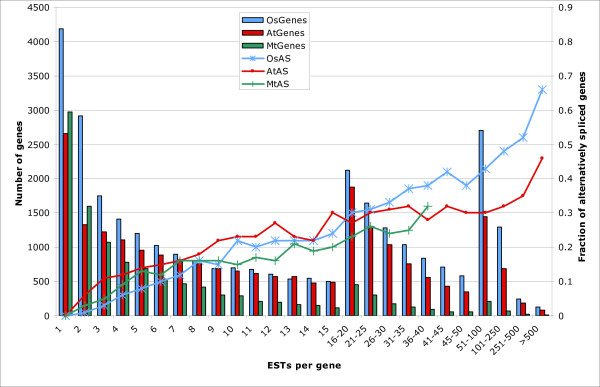
**Correlation between AS frequency and EST coverage**. The x-axis indicates groups of genes with certain numbers of ESTs. The primary y-axis for the bar graph indicates total number of genes within each group. The secondary y-axis for the line graph indicates the fraction of alternatively spliced genes for the group. Note that different bin sizes were used to keep the number of genes in each group greater than 500 in *At *and *Os*. AS data from groups with fewer than 80 genes in *Mt *were removed to reduce noise. *Lj *data were not shown as only the first six groups have more than 80 genes.

To compare the frequency of alternative splicing between different species, earlier studies relied on 10 randomly selected ESTs per gene as a basis for estimating AS frequency [4]. Here, only a small fraction (10–20%) of legume genes were covered by 10 or more ESTs, so this approach was not practical. Instead, we plotted the AS frequency for all groups of genes with similar EST coverage in different species, as shown in Figure 2. *Mt *categories with fewer than 80 genes total were removed to reduce noise due to small sample size, and *Lj *data are not included at all, as sample size was uniformly too small. When analyzed in this way, the fractions of alternatively spliced genes are similar regardless of species for nearly all size classes. For genes with four ESTs (the median EST number per gene in *Mt*), the observed AS frequency is 6–12% in *Mt*, *At*, and *Os *alike. For genes with nine to 11 ESTs (the median EST number per gene in *Os *and *At*), 15–23% are alternatively spliced. In general, the fraction of alternatively spliced genes keeps increasing with increasing transcript coverage, eventually reaching 66% in *Os *and 46% in *At *for genes with hundreds of ESTs, a levels similar to those observed in mammals [38,39]. Interestingly, the AS level in *Os *is consistently over 10% higher than in *At *in genes with more than 40 supporting ESTs.

### IntronR is the most abundant AS type in legumes

As shown in Table 2, the proportions of different AS types are similar in *Mt*, *At *and *Os*. (*Lj *data are also listed but are not included in the analysis as only ~100 AS events were identified). More than half of AS events in plants are IntronR, 6–11% are ExonS, and the remaining 30–40% involve different splice sites (AltD/A/P). These numbers are quite similar to those observed previously [1]. *Mt *has a slightly lower ratio of IntronR (51%) and a higher ratio of AltD (13%) compared with *At *and *Os*. Different levels of EST coverage have little effect on the composition of AS events. As shown in Additional file 1 (Supplementary Figure S4), the ratios of different AS types remain largely constant across all EST levels, particularly in *At *and *Os*. IntronR is the most abundant at all levels, with a relatively lower ratio in *Mt*. The ExonS ratio is consistently lower in *At *than in *Os *(and *Mt*), while the AltA ratio is higher.

**Figure 3:**
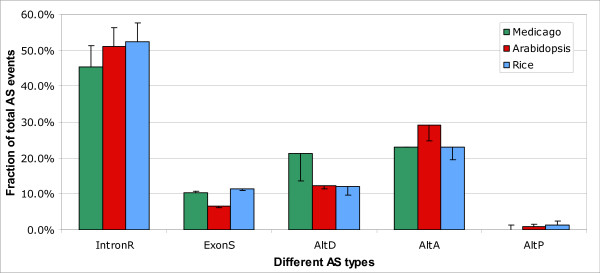
**Ratio of different AS types in a reliable subset of AS events**. The reliable data set consisted of AS events with multiple supporting ESTs for each isoform. IntronR is still the most abundant AS type in the subset. The error bar represents the ratio for each AS type in full data set described in Table 2.

To minimizes false AS events caused by sequencing errors or contaminations in the EST collection, we repeated the above analysis for the subset of AS events that are supported by at least two transcript sequences [40]. As shown in Figure 3, the ratio of IntronR decreased ~5% in all plants in this subset. *Mt *has the lowest ratio of IntronR (45%), 6–7% lower than in *At *and *Os*. The ratio of ExonS remains unchanged compared with the full data set. In *Mt *and *Os*, 10–11% AS events are ExonS compared to 7% in *At*. The AltD ratio in *Mt *increased significantly to 21% in the subset, nearly double the ratio in *At *and *Os*. In *At*, the AltA ratio is ~30% compared to 23% in *Mt *and *Os*. Similar tendencies were observed for subset data with even more transcripts supporting each isoform. Both the full and subset data indicate that *Mt *has a lower ratio of IntronR and a higher ratio of AltD, and that *At *has a lower ratio of ExonS but a higher ratio of AltA.

### Cross-species EST alignment in Medicago reveals hundreds of novel AS events

Even "reliable" AS events (as defined above) may not necessarily be functional. Because conservation is usually a good indicator of function, we deployed a cross-species approach similar to large-scale methods used previously in mammals to identify functional AS events [7,9]. All available EST sequences from *Lj*, *Gm*, and other legume species were aligned against *Mt *BACs. One concern with the cross-species approaches has been a potentially high error rate [7]. Here, even using an identity cutoff as high as 80%, hundreds of AS events were identified from either GeneSeqer or GMAP alignments alone, with approximately 40% of events consistent between the programs. Our analysis used only common events identified by the two programs to reduce false positive events from alignment errors. As shown in Table 3, 10–20% of the non-*Mt *legume transcript sequences could be mapped to *Mt *BACs and clustered to a total of 7,896 non-redundant genes, 81% of which have also *Mt *EST support. Approximately 70% of the introns identified from cross-species EST alignments were consistent with *Mt *EST supported introns. The gene structures derived from cross-species ESTs and *Mt *ESTs alignments were mostly consistent, demonstrating the value of cross-species ESTs in genome annotation [10]. In this analysis, a total of 307 *Mt *genes (3.9%) were found to be alternatively spliced, with 248 genes having no evidence of AS from *Mt *ESTs alone. If these novel AS events are included, the estimated frequency of *Mt *alternatively spliced gene increases from 9.6% to 10.4%. Interestingly, many more AS events were identified from soybean ESTs than from *Lj *ESTs, despite the similar evolutionary distance between *Mt-Gm *versus *Mt*-*Lj*. *At *and *Os *EST sequences were also applied in a comparable cross-species analysis, but only 1% of them could be mapped using the same criteria. No reliable AS events were deduced from *At *and *Os *transcript sequences.

**Table 3:** Cross-species EST alignments in Medicago

Species	EST/cDNA	Mapped to *Mt *BACs	Genes	Genes without *Mt *EST	AS Genes	Novel AS*	Predicted introns	Consistent introns^
Lotus	150,855	15,542 (10.3%)	2,955	367 (12.4%)	12 (3.3%)	8	5,606	4,256
Soybean	359,834	42,665 (11.9%)	5,810	925 (15.9%)	242 (4.2%)	201	16,758	11,420
Other legumes	127,684	26,547 (20.8%)	5,335	700 (13.1%)	69 (1.3%)	50	13,052	9,926

Total	638,373	84,754 (13.3%)	7,896	1,475 (18.7%)	307 (3.9%)	248	23,179	15,506

* Novel AS gene indicates genes not identified as alternative splicing by *Mt *EST.

^ Consistent introns indicate number of introns predicted from cross-species ESTs which are also supported by *Mt *EST.

Altogether, 367 cross-species AS events were identified from legume cross-species EST alignment, including 35.7% IntronR, 16.9% ExonS, 16.1% AltD, 29.1% AltA, and 2.2% AltP (Table 4). Compared with AS events identified using *Mt *ESTs alone, the cross-species AS events display a relatively lower ratio of IntronR and higher ratios of ExonS, AltD, and AltA. As most of the cross-species AS events are likely conserved between *Mt *and the native species of the EST, the ratio of each AS type in cross-species AS events could be interpreted to represent the ratio of functional AS events. However, the ratio of IntronR could have been underestimated by cross-species EST alignments because intron sequences are not as well-conserved as exons, even in closely related species. Thus, some cross-species ESTs retaining introns from their native species might have been filtered by the 80% identity cutoff. The location and outcome of cross-species AS events and same-species AS events are compared in Additional file 1 (Supplementary Table S2).

**Table 4:** AS events predicted from cross-species EST alignment in Medicago

Species	AS events	AltD	AltA	AltP	ExonS	IntronR
Lotus	12	2 (16.7%)	6 (50.0%)	1 (8.3%)	2 (16.7%)	1 (8.3%)
Soybean	276	40 (14.5%)	75 (27.2%)	5 (1.8%)	53 (19.2%)	103 (37.3%)
Other legume	87	20 (23%)	26 (29.9%)	2 (2.3%)	7 (8.0%)	32 (36.8%)

Total	367	59 (16.1%)	107 (29.1%)	8 (2.2%)	62 (16.9%)	131 (35.7%)

Approximately 90% of cross-species AS events are located in open reading frames (ORFs), much higher than the fraction (70–75%) in same-species AS events. There seem to be more cross-species and same-species AS events in the 5'-UTR than in the 3'-UTR (data not shown and [1]). For AS events in ORFs, the fractions of translation-readthrough events, where some amino acids are added to or removed from the protein without changing the reading frame, are similar (20–24%) in cross-species and same-species events. AltA has the highest translation-readthrough ratio (35–40%), and IntronR has the lowest (2–10%). Intriguingly, the ratio of AS events producing substrates for nonsense-mediated decay (NMD) [41] is higher in cross-species AS events than in same-species AS events. Nearly half of the cross-species AS events produce NMD substrates, compared with 30–40% in same-species AS events.

### Conserved AS events identified from cross-species EST alignments in legumes

To identify AS events with direct evidence of conservation in multiple species, two approaches were employed: (1) Align all legume ESTs to *Lj *TACs to identify conserved AS events predicted by the same ESTs between *Mt *and *Lj*; (2) Identify conserved AS events in *Mt *with EST evidence from multiple legume species, all showing the same AS pattern. A total of 242 AS events conserved between *Mt *and *Lj *were identified through method (1), including 92 (38.0%) IntronR, 26 (10.7%) ExonS, 78 (32.2%) AltA, 41 (17.0%) AltD, and 5 (2.1%) AltP events. These AS events are viewable at the ASIP website. Method (2) identified 22 completely conserved AS events in *Mt *(see Additional file 1, Supplementary Table S3). Nine of the 22 genes also have *At *and/or *Os *close homologs sharing the same AS pattern. For instance, *Mt *hypothetical protein AC156627_1 has both soybean and *Mt *ESTs support for an AltA event in the first ORF intron, whereby an isoform utilizes an alternative acceptor site 5-nt upstream (AACAG) of the constitutive acceptor site (AGCAG), producing a substrate possibly subject to NMD. *At *homologs (At5g25360.1 and At1g15350.1) and *Os *homolog (LOC_Os02g10720) both have exactly the same AS pattern, including the alternative acceptor sites. This gene seems to be plant-specific, as non-plant homologs can not be identified. Another example of completely conserved AS events is the *Mt *AP2 domain containing protein AC151460_3, where the 3'-UTR intron can be retained. One *At *homolog and three *Os *homologs also have the same intron retained. There are also some AS events conserved in legumes but not observed in *At *and *Os*. One example is AC124951_11, a highly expressed carbonic anhydrase gene with the 3'-UTR intron alternatively spliced (AltD) in legumes species. The AltD event is conserved in all legume species (*Mt*, *Lj*, *Gm*, and others), but not in *At *and *Os *even though hundreds of ESTs exist, indicating that this AS event is probably legume-specific.

**Figure 4:**
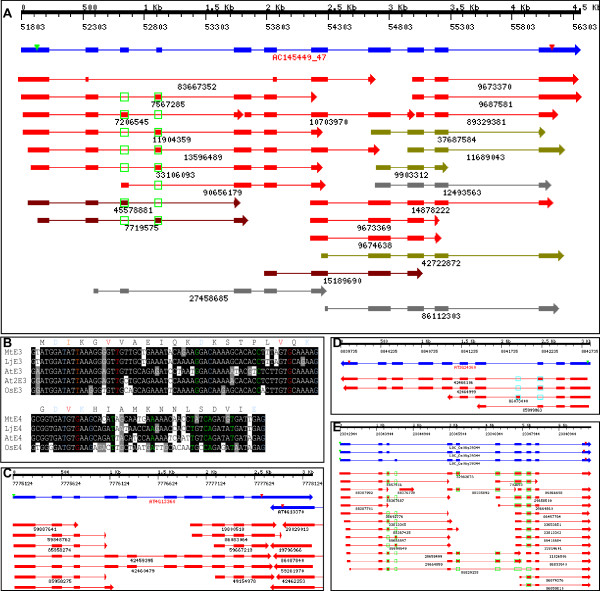
**Completely conserved ExonS event in plant enoyl-CoA hydratase/isomerase genes**. **A**: same-species and cross-species EST alignments in *Mt *gene locus AC145499_47. Filled boxes and arrows indicate exons, and lines indicate introns. Green open or filled boxes indicate exons skipped or retained in certain ESTs. The top black scale indicates coordinates for the gene locus on BAC (AC145499). The blue bar represents the IMGAG annotated gene model, with the green triangle representing the protein translation start codon and the red triangle representing the stop codon. Red bars represent individual same species EST alignments. Purple bars represent *Lj *ESTs, dark yellow bars represent soybean ESTs, and gray bars represent ESTs from other legume species. **B**. Multiple sequence alignments of the mutual exclusive exons. E3 indicates the Exon 3 and E4 indicates the Exon 4. At2E3 refers to the exon in the second copy of *At *gene (At4g13360). Amino acids encoded by *Mt *sequences are list at the top of sequence alignment. Degenerate positions (change in nucleotide will not change amino acids) which are conserved in all exons are highlighted in colors. **C**. EST alignment in the second copy of *At *gene (At4g13360). Only exon E3 exists in this gene and no ExonS can be detected. **D, E**. EST alignment in *At *and *Os *genes where the ExonS pattern is completely conserved.

One example of a completely conserved ExonS event occurs in an enoyl-CoA hydratase/isomerase gene (*Mt*: AC145449_47). As shown in Figure 4A, the IMGAG-annotated gene structure for AC145449_47 contains 11 exons, each with strong EST support. Exon3 (65 nt) and Exon4 (53 nt) are mutually exclusive. In one isoform, Exon3 is retained and Exon4 is skipped (*Mt*: 7206545, 90656179; *Lj: *45578881; Lupine: 27458685). In another isoform, Exon4 is retained with Exon3 skipped (*Mt*: 7567285, 11904359, 13596489, 33106093; *Lj*: 7719575). The two mRNA isoforms therefore encode two proteins (418 aa and 414 aa) differing slightly in their predicted Enoyl-CoA hydratase domain (ECH, pfam00378). No isoform contains both exons, while it is possible to skip both (*Mt*: 83667352). Two genes in *At *(At4g13360 and At3g24360), one gene in *Os *(LOC_Os06g39344) and one in *Lj *(*Lj*TC_2465, AP006370.1: 88858–94512) are the closest homologs to AC145449_47. Exactly the same AS pattern was observed in all the homologous genes except for At4g13360, where the 65-nt exon (Exon3) was retained constitutively and no trace of the 53-nt exon can be found in the corresponding region (Figure 4C–E). Sequence comparison revealed several nucleotide bases in degenerate codons conserved in all four species (Figure 4B). These bases may contribute to the recognition of (or skipping) the exon.

## Discussion

### Comparison of AS frequencies in different species

In this study, alignment of current EST and genomic sequences revealed that ~10% of expressed genes are alternatively spliced in *Mt *compared with 20% in *At *and *Os*. This difference is mainly due to the lower EST coverage found in *Mt*. We demonstrated that the AS frequencies in the three plants are essentially similar when adjusted for genes having comparable EST numbers. This conclusion is different from the conclusion drawn in a recent study based on EST pairs gapped alignments, in which a greater degree of variation was observed for different plant species [3]. Interpretation of EST-only data can be confounded by extensive gene duplication events. With more plant genome sequences becoming available, it should soon be possible to more precisely address the intriguing questions concerning the extent and evolution of AS in plants.

Alternatively spliced isoforms are usually in low abundance, the chance of capturing them in a small EST collection is low, making it difficult to estimate AS frequencies accurately. Supposing a functional event has certain percentage *p *of transcripts alternatively spliced, the probability of observing an AS event with *n *ESTs covering the alternative splice site is 1 - (1 - *p*)^*n*^. For example, if an alternatively spliced isoform were generated *p *= 10% of the time, n = 10 transcript sequences would give a 65% probability of observing this event, and 22 transcript sequences would be required to have >90% probability of observing the event. Our results show that the AS frequency for genes with small numbers of ESTs are similar in *Mt*, *At*, and *Os*, suggesting that they all have similar levels of functional AS events.

In cases where AS isoforms are even lower in abundance, greater numbers of transcripts would be clearly necessary to detect the event. Nevertheless, *Os *seems to have a higher frequency of AS in genes with >30 ESTs than either *Mt *or *At*. Focusing on genes with >40 ESTs only, the AS frequency in *Os *is consistently (>10%) higher than in *At*. For this analysis, we did not include transcripts from *Os *subspecies *indica *in order to eliminate the possibility that the higher AS frequency is falsely caused by cross-subspecies ESTs. In any case, the error rates from EST sequencing or genome contamination are probably similar in all three plants. Consequently, *Os *does seem to have higher levels of low-abundance AS events than *At *(or *Mt*). Some of the low-abundance events may be splicing errors captured in EST libraries constructed from plant tissues under various growth conditions, so the higher level of low-abundance AS events in *Os *could indicate higher error rates for the *Os *spliceosome.

Not surprisingly, observed AS frequency is highly correlated with EST numbers in all three plants. Highly expressed genes (genes with large numbers of ESTs) are more likely to be detected as alternatively spliced. Over 60% and 40% genes with more than 500 ESTs are alternatively spliced in *Os *and *At*, respectively. This is comparable to the level in human [42]. Half of human genes are alternatively spliced by the criterion that AS isoforms occurs in at least 1% of the observed transcripts, but only 20% of human genes are alternatively spliced if the required abundance level is increased to >10% [42]. This frequency is notably similar to the frequency in plants under the same abundance level, suggesting that the frequency of regulated AS events in plants may not be significantly lower than in mammals.

### Splicing errors and functional AS events

A clear difference between AS in plants and mammals is the predominance of IntronR in plants and ExonS in mammals. Both model legumes, *Mt *and *Lj*, have 40–50% of AS events as IntronR, a level noticeably lower than in *At *and *Os*, but still much higher than in mammals. Similar to the situation in *At *and *Os *[1], introns shorter than 70 nt are more likely to be retained in legumes (data not shown). The spliceosome is a large dynamic RNA-protein complex involving hundreds of proteins. If an intron is too small, the assembly and structure transformation of spliceosome will be constrained and may lead to inefficient splicing and IntronR [1]. As the size of introns is considerably larger in *Mt *and *Lj*, fewer introns will be retained due to steric hindrance, possibly leading to a lower frequency of IntronR in legumes. These data also suggest that some AS events may be splicing errors. As we proposed in [1], the most common splicing error in plants is probably a failure to recognize and splice out introns, so IntronR should be the most common AS type. In mammals, where introns are defined through an exon recognition mechanism, a failure to recognize some exons, and therefore skip them, is likely the most common error. Consequently, ExonS is the most common AS type in human.

Observed AS events are a mixture of functional AS events and splicing errors. Other types of error, such as sequencing errors, genome contamination, and alignment errors, will also contribute to the predicted level of AS events. Two alignment programs (GeneSeqer and GMAP) were applied and only common AS events were used in this study to minimize alignment errors. Genome contamination could be minimized by elimination of ESTs retaining all predicted introns. Distinguishing functional AS events from splicing errors, however, is not an easy task. We attempted to achieve this goal by two methods. First, we selected AS events with each isoform supported by multiple transcripts. As splicing errors are expected to occur at low frequency, the chances they will be captured in two distinct transcripts are low. In this data set, the frequency of IntronR is slightly lower, but still the highest among the five AS types, indicating that IntronR is indeed the most abundant regulated AS result. The second method is to look for conserved AS events through cross-species EST comparison and orthologous gene comparison. A few AS events were completely conserved in *Mt*, *Lj*, *At *and *Os*.

Functional AS events, however, may not always be conserved. As a dynamic process, splicing requires hundreds of proteins as well as some snRNAs to function accurately [14]. Mutations in both *trans*- and *cis*-elements on target genes will impact splicing patterns. Depending on when the mutation and fixation event occurs, functional AS events can be shared among closely related species or be lineage-specific. The AltD event in 3'-UTR of the highly expressed carbonic anhydrase gene (AC124951_11) may be a good example shared by legume species. Lineage-specific functional AS events are difficult to define from EST data alone.

### Centralized data place and standard data set for ASIP

As more plant genomes and ESTs are being sequenced, more AS events will be identified in the future. It is important to have a centralized place to store and compare all AS data. In animal systems, a comprehensive database, ASAP [43] includes AS data from 16 sequenced animals, which makes a comparison across different animal species straightforward. Such a database is also needed in plants, as the study of splicing signals and alternative splicing are just starting. The AS data identified in this study have been deposited in the ASIP database at PlantGDB [37], where previous AS data are stored and can be easily compared [1]. Moreover, a database collecting genes related to splicing in *At*, animals and yeast is available through the SRGD database at PlantGDB [14,44]. In the future, the database will be expanded to *Os *and other sequenced plant genomes including *Mt*, *Lj *and poplar. The analysis programs and plant genome browsers available at PlantGDB should facilitate the deep mining of AS data in plants. A core data set in which the AS events are conserved in all sequenced plants will be extremely useful for understanding the function of AS events, as well as the signals and regulation of this important and intriguing phenomenon.

## Conclusion

As in *At *and *Os*, AS events are also widespread in the two model legumes *Mt *and *Lj*. Thousands of AS events were identified in *Mt *through a combination of same- and cross-species EST alignments. The frequency of alternatively spliced genes is similar across different plant species when the number of ESTs is standardized. Compared with mammals, plants are thought to have a relatively low frequency of alternatively spliced genes. Our results indicate that this assessment may be due in part to the comparatively low EST coverage in plant species. Among all five AS types discussed, IntronR is the most abundant in different subsets of genes, as previously observed in *At *and *Os*. We also identified hundreds of novel and conserved AS events through cross-species ESTs alignments. This is the first study in plants using cross-species ESTs to explore AS. For species with large EST collections but scant genome sequence data, including wheat and barley, aligning their ESTs to a closely related reference genome, such as *Os*, should shed light on alternative splicing in these species.

## Methods

### Data sets

The *Medicago *Genome Sequence Consortium (MGSC) release 1.0, consisting of the 1,826 BACs analyzed in this study, were downloaded from *Medicago *genome sequencing project website [45]. The assembly comprises a total of 186.2 Mb of non-redundant genome sequence, an estimated 38–47% of the entire genome and 55–58% of total gene space [46]. All other sequence data sets used in this study were current as of July 17, 2006, the cutoff date for BACs incorporated into the Mt1.0 genome assembly. For *Lotus japonicus*, 1,394 BAC/TACs were downloaded from the NCBI [47] nucleotide database using the query "txid34305 [ORGN:noexp] AND HTG [KYWD]". *Arabidopsis *genome sequences and gene annotation (TAIR release 6.0) were downloaded from the GenBank FTP site [48], and rice genome sequences and gene annotation (TIGR release 4.0) were downloaded from the TIGR FTP site [49].

All EST sequences (including full-length cDNAs) were retrieved from GenBank nucleotide database. Sets of 225,920 *Mt *and 150,855 *Lj *transcript sequences were collected using the queries (txid3880 [ORGN] AND "biomol mrna" [PROP]) and (txid34305 [ORGN] AND "biomol mrna" [PROP]), respectively. Soybean transcript sequences (359,834) were retrieved using the query (txid3847 [ORGN] AND "biomol mrna" [PROP]), and 127,684 transcript sequences from all other legumes were retrieved by using the query (txid3803 [ORGN:exp] NOT txid3880 [ORGN] NOT txid34305 [ORGN] NOT txid3847 [ORGN] AND "biomol mrna" [PROP]). For *At*, 691,516 transcript sequences were retrieved using the query (txid3702 [ORGN] AND "biomol mrna" [PROP] AND srcdb_ddbj/embl/genbank [PROP]). For *Os*, 1,009,574 ESTs from the *japonica *cultivar-group were retrieved using query (txid39947 [ORGN] AND "biomol mrna" [PROP] AND srcdb_ddbj/embl/genbank [PROP]). We intentionally excluded transcript sequences from the *indica *cultivar-group to reduce possible false positive alignments caused by differences between the two *Os *cultivar-groups.

### Spliced alignment of transcript to genome sequences

The legume transcript sequences were mapped to the *Mt *and *Lj *BAC sets using the two computer programs GeneSeqer [34] and GMAP [35]. The splice site models for GeneSeqer were set to *Medicago*-specific parameters using the program option "-s Medicago". Default parameters were used for all other options. Default alignment parameters were used for GMAP. For *At *and *Os*, only GMAP alignments were performed locally, and GeneSeqer alignments derived from a larger data set were downloaded from PlantGDB [50].

GMAP and GeneSeqer output alignment files were processed by a pipeline (ASpipe1.0, available through SourceForge [51]) developed from Perl and shell scripts used in a previous study [1]. ASpipe extracts coordinates and scores for high-quality intron/exon/alignments from the original program outputs and stores them in MySQL5.0 databases. For same-species EST alignments, the criteria for high-quality alignments were >95% sequence identity and >80% coverage (defined as the portion the transcript sequence aligned to the genomic sequence). The high identity (95%) cutoff minimizes false mapping of transcript sequences to incomplete genomes. For cross-species transcript alignments, the identity cutoff was decreased to 80%, which selects reliable alignments from divergent transcript sequences. Redundant EST alignments in *Mt *were removed by comparison with the non-redundant gene list provided for Mt1.0 [33]. Exons mapped with >95% and >80% sequence identity were considered as reliably identified exons for same-species and cross-species mappings, respectively. Introns with reliable neighboring exons on both ends were considered as reliably identified introns. A transcription unit (TU) was defined as a consecutive genomic region where transcript sequences were mapped and clustered. Annotated gene models may contain multiple TUs. For *Mt*, *At *and *Os*, annotated genes were used as the base for analysis. For *Lj*, where no gene annotation is available, TUs were the base for analysis.

### Identification of alternative splicing (AS) and conserved AS events

The coordinates of reliable introns and exons were compared in a pairwise fashion in order to identify candidates for AS events. For intron/intron comparison, if two introns had the same 3'-end but a different 5'-end, this event was classified as AltD. If two introns differed only in the 3'-ends, this event was classified as AltA. AltP events refer to introns overlapping with each other but with both 5'- and 3'-ends differing. For intron/exon comparisons, if an intron was completely covered by an exon, the event was classified as IntronR. If an exon was completely covered by an intron, the event was classified as ExonS. ExonS events involving terminal exons and the AltA/D/P events related to ExonS events were removed. The process and algorithm for identifying and analyzing AS events is described in more detail in [1]. AS events identified from cross-species EST alignment were labeled as "cross-species AS events". Correspondingly, the events from same-species EST alignment were referred to as "same-species AS events".

Conserved AS events were identified in two ways: (1) Comparing cross-species AS events with same-species AS events and other cross-species AS events from different species; (2) Identifying orthologous gene pairs between *Mt *and *Lj *and comparing their AS events. In the first method, the *Mt *genome coordinates of the AS events predicted from multiple species ESTs were compared. Only events with identical coordinates of an alternatively processed intron(s)/exon(s) were regarded as completely conserved. In the second method, the orthologous genes were identified by searching ESTs mapped in both *Mt *and *Lj *genomes. In some cases, orthologs in *At *and *Os *were identified by reciprocal BLAST using annotated protein sequences from *At*, *Mt *and *Os*. Gene structures and AS events of orthologous genes were then compared to identify conserved AS events.

## Abbreviations

AltA, Alternative Acceptor site; AltD, Alternative Donor site; AltP, Alternative Position (both donor and acceptor sites are different). AS, Alternative Splicing; *At, Arabidopsis thaliana*; EST, expressed sequence tag; ExonS, Exon Skipping; IntronR, Intron Retention; *Lj*: *Lotus japonicus*; *Mt: Medicago truncatula*; NMD, nonsense-mediated decay; ORF, open reading frame; *Os, Oryza sativa;*

## Authors' contributions

BBW conceived of the study, performed research, analyzed data and drafted the manuscript. MOT participated in data analysis and web page creation. VB participated in data analysis and presentation and helped to draft the manuscript. NDY participated in the design of this study, coordinated data analysis and helped to draft the manuscript. All authors read and approved the final manuscript.

## Supplementary Material

Additional file 1 **Supplementary figures and tables**. This pdf document contains supplementary figures and tables for the main manuscript. Click here for file

## References

[B1] Wang BB, Brendel V (2006). Genomewide comparative analysis of alternative splicing in plants. Proc Natl Acad Sci USA.

[B2] Campbell MA, Haas BJ, Hamilton JP, Mount SM, Buell CR (2006). Comprehensive analysis of alternative splicing in rice and comparative analyses with Arabidopsis. BMC Genomics.

[B3] Ner-Gaon H, Leviatan N, Rubin E, Fluhr R (2007). Comparative cross-species alternative splicing in plants. Plant Physiol.

[B4] Kim E, Magen A, Ast G (2007). Different levels of alternative splicing among eukaryotes. Nucleic Acids Res.

[B5] Gupta S, Zink D, Korn B, Vingron M, Haas SA (2004). Genome wide identification and classification of alternative splicing based on EST data. Bioinformatics.

[B6] Ner-Gaon H, Fluhr R (2006). Whole-genome microarray in Arabidopsis facilitates global analysis of retained introns. DNA Res.

[B7] Kan Z, Castle J, Johnson JM, Tsinoremas NF (2004). Detection of novel splice forms in human and mouse using cross-species approach. Pac Symp Biocomput.

[B8] Sugnet CW, Kent WJ, Ares M, Haussler D (2004). Transcriptome and genome conservation of alternative splicing events in humans and mice. Pac Symp Biocomput.

[B9] Chen FC, Chen CJ, Ho JY, Chuang TJ (2006). Identification and evolutionary analysis of novel exons and alternative splicing events using cross-species EST-to-genome comparisons in human, mouse and rat. BMC Bioinformatics.

[B10] Zhu W, Buell CR (2007). Improvement of whole-genome annotation of cereals through comparative analyses. Genome Res.

[B11] Chen FC, Wang SS, Chaw SM, Huang YT, Chuang TJ (2007). Plant Gene and Alternatively Spliced Variant Annotator. A plant genome annotation pipeline for rice gene and alternatively spliced variant identification with cross-species expressed sequence tag conservation from seven plant species. Plant Physiol.

[B12] Reddy AS (2007). Alternative Splicing of Pre-Messenger RNAs in Plants in the Genomic Era. Annu Rev Plant Biol.

[B13] Reddy ASN (2001). Nuclear pre-mRNA splicing in plants. Critical Rev Plant Sci.

[B14] Wang BB, Brendel V (2004). The ASRG database: identification and survey of Arabidopsis thaliana genes involved in pre-mRNA splicing. Genome Biol.

[B15] Palusa SG, Ali GS, Reddy AS (2007). Alternative splicing of pre-mRNAs of Arabidopsis serine/arginine-rich proteins: regulation by hormones and stresses. Plant J.

[B16] Kalyna M, Lopato S, Voronin V, Barta A (2006). Evolutionary conservation and regulation of particular alternative splicing events in plant SR proteins. Nucleic Acids Res.

[B17] Iida K, Go M (2006). Survey of conserved alternative splicing events of mRNAs encoding SR proteins in land plants. Mol Biol Evol.

[B18] Isshiki M, Tsumoto A, Shimamoto K (2006). The serine/arginine-rich protein family in rice plays important roles in constitutive and alternative splicing of pre-mRNA. Plant Cell.

[B19] Gupta S, Wang BB, Stryker GA, Zanetti ME, Lal SK (2005). Two novel arginine/serine (SR) proteins in maize are differentially spliced and utilize non-canonical splice sites. Biochim Biophys Acta.

[B20] Gao H, Gordon-Kamm WJ, Lyznik LA (2004). ASF/SF2-like maize pre-mRNA splicing factors affect splice site utilization and their transcripts are alternatively spliced. Gene.

[B21] Wang BB, Brendel V (2006). Molecular characterization and phylogeny of U2AF35 homologs in plants. Plant Physiol.

[B22] Golovkin M, Reddy AS (1996). Structure and expression of a plant U1 snRNP 70K gene: alternative splicing of U1 snRNP 70K pre-mRNAs produces two different transcripts. Plant Cell.

[B23] Gupta S, Ciungu A, Jameson N, Lal SK (2006). Alternative splicing expression of U1 snRNP 70K gene is evolutionary conserved between different plant species. DNA Seq.

[B24] Zhou Y, Zhou C, Ye L, Dong J, Xu H, Cai L, Zhang L, Wei L (2003). Database and analyses of known alternatively spliced genes in plants. Genomics.

[B25] Ramos J, Clemente MR, Naya L, Loscos J, Perez-Rontome C, Sato S, Tabata S, Becana M (2007). Phytochelatin synthases of the model legume Lotus japonicus. A small multigene family with differential response to cadmium and alternatively spliced variants. Plant Physiol.

[B26] Szczyglowski K, Kapranov P, Hamburger D, de Bruijn FJ (1998). The Lotus japonicus LjNOD70 nodulin gene encodes a protein with similarities to transporters. Plant Mol Biol.

[B27] Horst I, Welham T, Kelly S, Kaneko T, Sato S, Tabata S, Parniske M, Wang TL (2007). TILLING Mutants of Lotus japonicus Reveal that Nitrogen Assimilation and Fixation can Occur in the Absence of Nodule-enhanced Sucrose Synthase. Plant Physiol.

[B28] Jeong SC, Yang K, Park JY, Han KS, Yu S, Hwang TY, Hur CG, Kim SH, Park PB, Kim HM, Park YI, Liu JR (2006). Structure, expression, and mapping of two nodule-specific genes identified by mining public soybean EST databases. Gene.

[B29] Hamada S, Ito H, Hiraga S, Inagaki K, Nozaki K, Isono N, Yoshimoto Y, Takeda Y, Matsui H (2002). Differential characteristics and subcellular localization of two starch-branching enzyme isoforms encoded by a single gene in Phaseolus vulgaris L. J Biol Chem.

[B30] Neumann P, Pozarkova D, Macas J (2003). Highly abundant pea LTR retrotransposon Ogre is constitutively transcribed and partially spliced. Plant Mol Biol.

[B31] Young ND, Cannon SB, Sato S, Kim D, Cook DR, Town CD, Roe BA, Tabata S (2005). Sequencing the genespaces of Medicago truncatula and Lotus japonicus. Plant Physiol.

[B32] Town CD (2006). Annotating the genome of Medicago truncatula. Curr Opin Plant Biol.

[B33] Medicago genome sequence release 1.0. http://www.medicago.org/genome/downloads/Mt1/.

[B34] Brendel V, Xing L, Zhu W (2004). Gene structure prediction from consensus spliced alignment of multiple ESTs matching the same genomic locus. Bioinformatics.

[B35] Wu TD, Watanabe CK (2005). GMAP: a genomic mapping and alignment program for mRNA and EST sequences. Bioinformatics.

[B36] Goodall GJ, Filipowicz W (1991). Different effects of intron nucleotide composition and secondary structure on pre-mRNA splicing in monocot and dicot plants. Embo J.

[B37] Alternative Splicing In Plants (ASIP). http://www.plantgdb.org/ASIP/.

[B38] Johnson JM, Castle J, Garrett-Engele P, Kan Z, Loerch PM, Armour CD, Santos R, Schadt EE, Stoughton R, Shoemaker DD (2003). Genome-wide survey of human alternative pre-mRNA splicing with exon junction microarrays. Science.

[B39] Brett D, Pospisil H, Valcarcel J, Reich J, Bork P (2002). Alternative splicing and genome complexity. Nat Genet.

[B40] Alexandrov NN, Troukhan ME, Brover VV, Tatarinova T, Flavell RB, Feldmann KA (2006). Features of Arabidopsis Genes and Genome Discovered using Full-length cDNAs. Plant Mol Biol.

[B41] Lewis BP, Green RE, Brenner SE (2003). Evidence for the widespread coupling of alternative splicing and nonsense-mediated mRNA decay in humans. Proc Natl Acad Sci USA.

[B42] Kan Z, States D, Gish W (2002). Selecting for functional alternative splices in ESTs. Genome Res.

[B43] Kim N, Alekseyenko AV, Roy M, Lee C (2007). The ASAP II database: analysis and comparative genomics of alternative splicing in 15 animal species. Nucleic Acids Res.

[B44] Splicing Related Genes Database (SRGD). http://www.plantgdb.org/SRGD.

[B45] Medicago genome sequencing project. http://www.medicago.org/genome/.

[B46] Medicago Genome Sequence Release 1.0 white book. http://www.medicago.org/genome/downloads/Mt1/Mt1.0.pdf.

[B47] National Center for Biotechnology Information (NCBI). http://www.ncbi.nlm.nih.gov/.

[B48] NCBI Arabidopsis Genome Sequence FTP Site. ftp://ftp.ncbi.nih.gov/genomes/Arabidopsis_thaliana/.

[B49] TIGR Rice Genome Sequences Release 4.0 FTP site. ftp://ftp.tigr.org/pub/data/Eukaryotic_Projects/o_sativa/annotation_dbs/pseudomolecules/version_4.0/.

[B50] Dong Q, Lawrence CJ, Schlueter SD, Wilkerson MD, Kurtz S, Lushbough C, Brendel V (2005). Comparative plant genomics resources at PlantGDB. Plant Physiol.

[B51] ASpipe project at SourceForge. https://sourceforge.net/projects/aspipe/.

