# Supplementary material for: Cross-species EST alignments reveal novel and conserved alternative splicing events in legumes
Source: BMC Plant Biol. 2008 Feb 19;8:17. doi: 10.1186/1471-2229-8-17 (PMC2277414; doi:10.1186/1471-2229-8-17)
Supplement: Additional file 1 — Supplementary figures and tables. This pdf document contains supplementary figures and tables for the main manuscript. [file 1471-2229-8-17-S1.pdf]

Additional document for

Wang *etc*, “Cross-species EST alignments reveal novel and conserved alternative splicing events in legumes”

I. Supplementary figures

Supplementary Figure S1. Five types of AS events and examples in Medicago.

Supplementary Figure S2. Distribution of GC-content in higher plant introns and exons.

Supplementary Figure S3. Distribution of base content in higher plant introns.

Supplementary Figure S4. Abundance of different alternatively splicing types change only slightly in groups with various EST levels.

II. Supplementary tables

Supplementary Table S1. Average base content and differences in plant introns and exons.

Supplementary Table S2. Outcome of AS events and cross-species AS events.

Supplementary Table S3. Conserved AS events identified from cross-species EST studies.

Supplementary Figure S1. Five types of AS events and examples in Medicago. The top black scale indicates coordinates for the gene locus on the genome. The blue bar represents IMGAG annotated gene model, with green triangle representing protein translation start codon and red triangle representing stop codon. Red bars represent individual EST alignment. Filled boxes and arrows indicate exons. Thin lines indicate introns. The green lines represent introns that may be retained or spliced. Green open or filled boxes indicate exons skipped or retained in certain ESTs. Vertical bars represent the alternative donor/acceptor sites. In the ASIP database, different donor and acceptor sites are denoted by different colors.

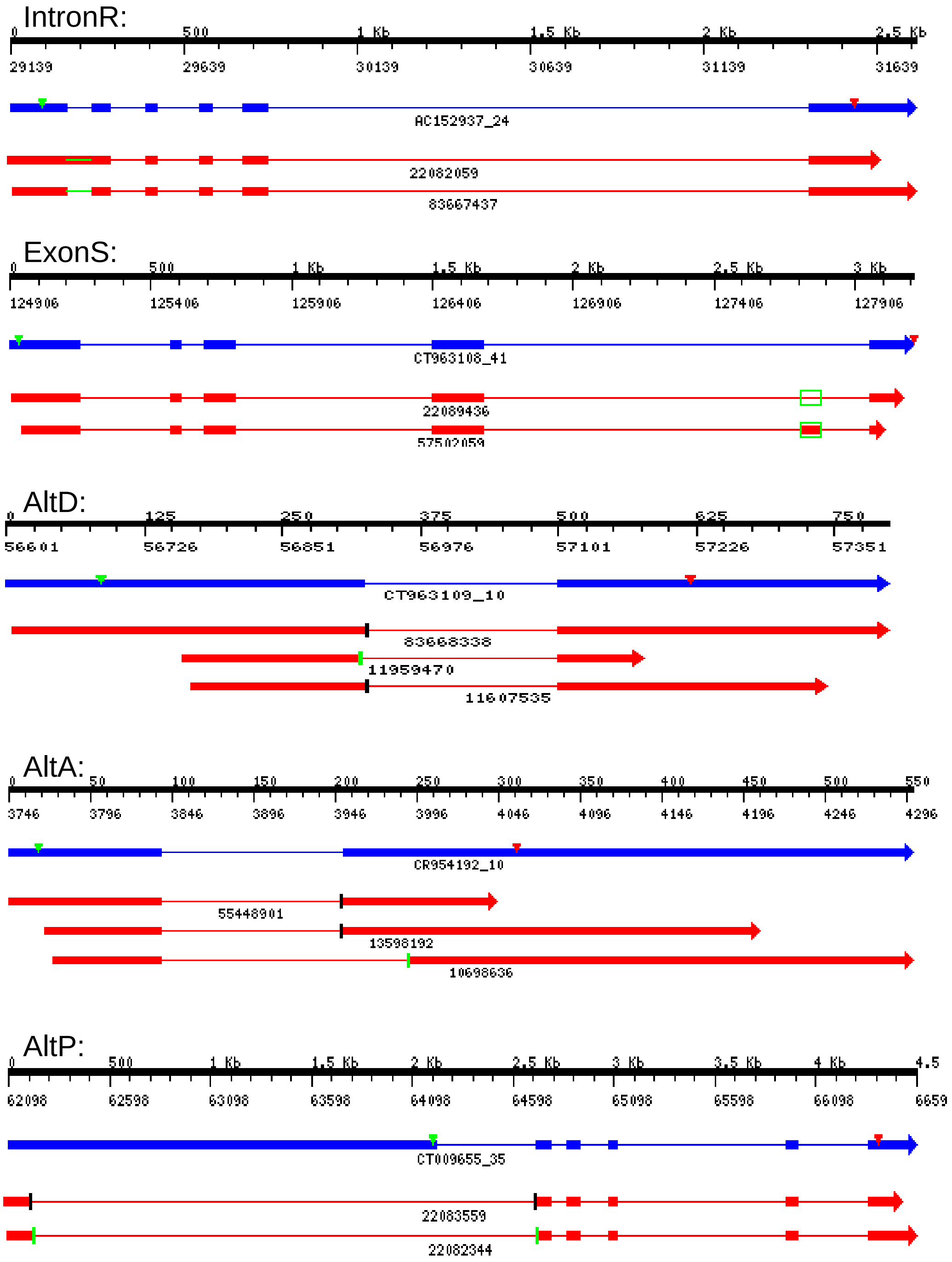

Supplementary Figure S2. Distribution of GC-content in higher plant introns and exons. The x-axis indicates the GC-content of either (A) introns or (B) internal exons. The bin size is 1%. y-axis indicates the fraction of total introns (A) or internal exons (B) for a given GC-content.

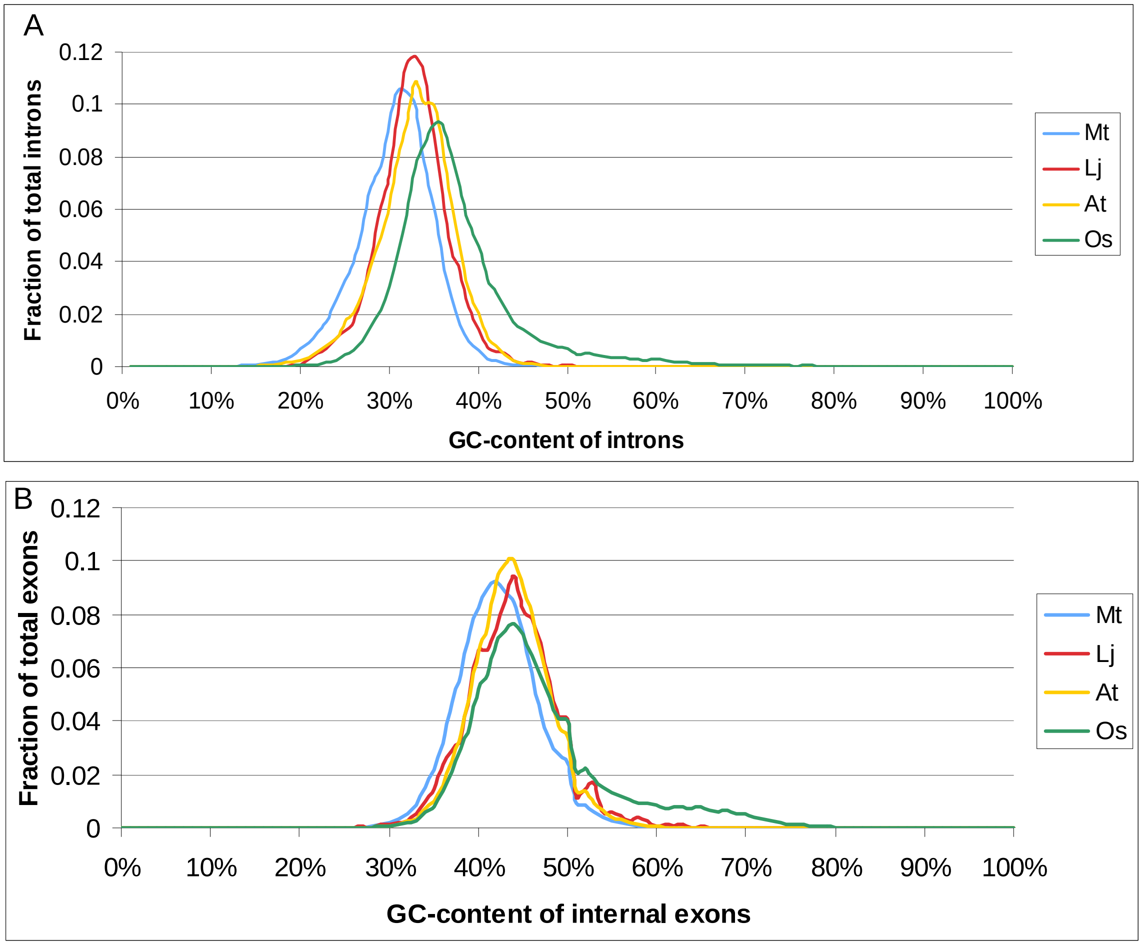

Supplementary Figure S3. Distribution of base content in higher plant introns. The x-axis indicates the content of base G, C, A, T in introns. The bin size is 1%. y-axis indicates the fraction of total introns for a given base content.

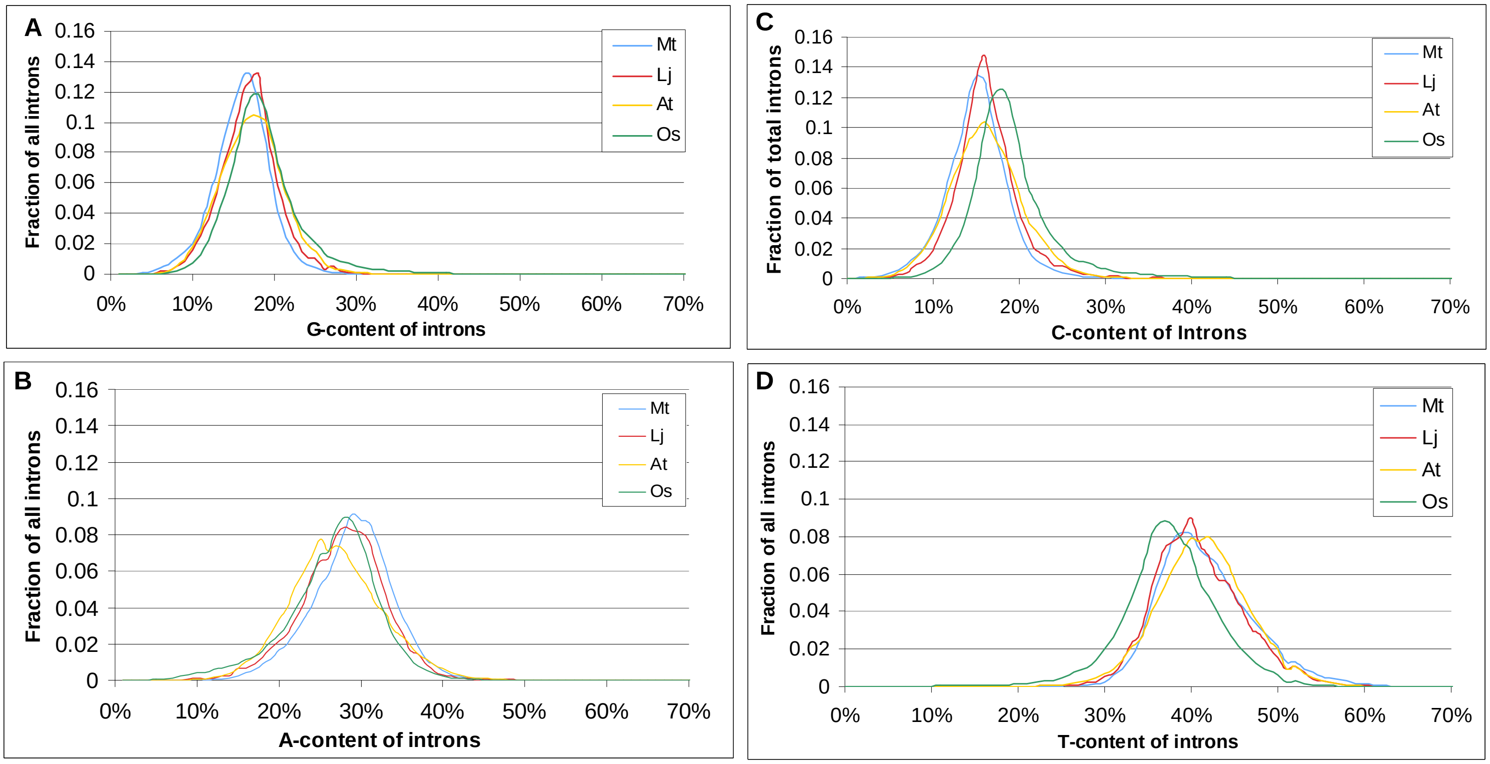

Supplementary Figure S4. Abundance of different alternatively splicing types change only slightly in groups with various EST levels. Groups with less than 50 AS events were removed to reduce noise. Each group has IntronR to be the most abundant AS type. The ExonS ratio (red bar) seems consistently higher in *Os* than in *At*, while AltA ratio (green bar) seems lower. In *At* and *Os*, the ratio of each AS type remains largely constant cross different EST level. *Mt* data have more variation due to the small number (<100) of AS events in most groups.

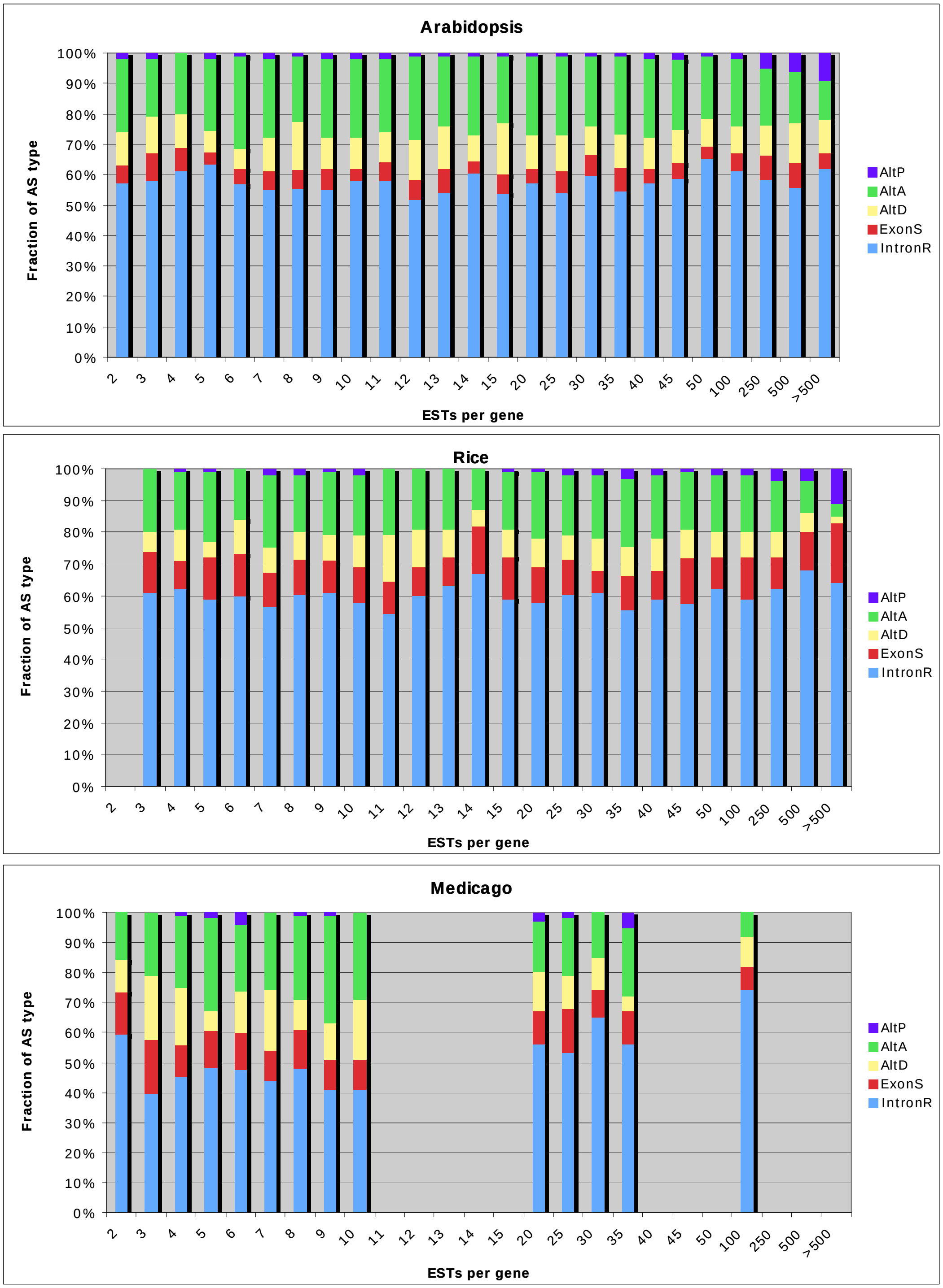

Supplementary Tables

Supplementary Table S1. Average base content and differences in plant introns and exons.

	Medicago		Lotus		Arabidopsis		Rice		Differences between exons and introns			
	Introns	Exons	Introns	Exons	Introns	Exons	Introns	Exons	*Mt*	*Lj*	At	Os
A	28.5%	29.6%	27.4%	28.7%	26.6%	29.0%	26.1%	27.5%	1.1%	1.3%	2.4%	1.4%
T	41.3%	28.6%	40.4%	27.8%	40.9%	27.6%	37.1%	26.0%	-12.7%	-12.6%	-13.3%	-11.1%
G	15.6%	23.4%	16.5%	24.3%	16.9%	23.8%	18.3%	25.2%	7.8%	7.8%	6.9%	6.9%
C	14.7%	18.5%	15.8%	19.1%	15.7%	19.5%	18.6%	21.3%	3.8%	3.3%	3.8%	2.7%
GC	30.2%	41.9%	32.3%	43.5%	32.6%	43.3%	36.9%	46.5%	11.7%	11.2%	10.7%	9.6%

Supplementary Table S2. Outcome of AS events and cross-species AS events. Gm-*Mt* indicates AS events identified by aligning Soybean ESTs against *Mt* BACs. ORF indicates the number of AS events located in protein coding region (open reading frame). Translate-through indicates the number of AS events will add/remove some amino acid residues to/from the wild type proteins without changing the reading frame. NMD indicates the number of AS events producing substrates subjecting to nonsense-mediated mRNA decay.

	ORF / all				Translate-through / ORF				NMD / all			
SItype	*Mt*	At	Os	Gm-*Mt*	*Mt*	At	Os	Gm-*Mt*	*Mt*	At	Os	Gm-*Mt*
AltD	72.4%	70.5%	59.3%	96.0%	22.9%	26.6%	22.8%	20.8%	40.0%	41.4%	30.2%	40.0%
AltA	75.6%	79.3%	72.0%	84.7%	36.5%	40.0%	34.0%	36.0%	38.0%	35.9%	30.1%	44.1%
AltP	100.0%	82.4%	87.6%	66.7%	0.0%	45.2%	28.2%	100.0%	50.0%	15.7%	25.8%	0.0%
ExonS	61.3%	70.0%	66.9%	96.9%	38.8%	30.6%	20.4%	35.5%	18.8%	35.6%	29.5%	43.8%
IntronR	71.0%	75.1%	69.4%	89.8%	6.5%	7.6%	10.1%	2.3%	43.8%	47.2%	34.6%	67.3%
Total	71.9%	75.5%	68.7%	89.9%	22.8%	22.2%	20.2%	24.5%	38.6%	41.9%	31.9%	49.4%

Supplementary Table S3. Conserved AS events identified from cross-species EST studies.

Mt Genes	Annotation	ESTgi	AS type	Legumes	Arabidopsis	Rice	Note
CT573215_20	Peptidase aspartic, active site IPR001461:Peptidase A1, pepsin IPR001969:Peptidase aspartic, active site IPR009007:Peptidase aspartic, catalytic	13783598	AltA	*Mt*,*Ot*			
CT573078_1	Protein of unknown function UPF0172 IPR005366:Protein of unknown function UPF0172	7239055	AltA	*Mt*,*Gm*			
AC156627_1	hypothetical protein	83668035	AltA	*Mt*	At5g25360, At1g15350	LOC_Os02g10720	AltA site conserved in all species (...AACAG|AGCAG)
AC137836_27	Actin/actin-like IPR004000:Actin/actin-like IPR004001:Actin	2604318	AltA	*Mt*,*Ot*		LOC_Os05g01600, LOC_Os11g06390	5'-UTR region 5nt in *Mt*, 9nt in *Os*; LOC_Os01g64630 (AltD)
AC123547_19	hypothetical protein	11904316	AltA	*Mt*,*Ot*			
AC167403_17	Dormancyauxin associated IPR008406:Dormancyauxin associated	6654880	AltD	*Mt*,*Gm*,*Ot*			
AC167330_3	IPR001680:WD-40 repeat IPR000009:Protein phosphatase 2A regulatory subunit PR55 IPR011046:WD40-like WD40-like::short desc	7796415	AltD	*Ot*	At1g17720, At1g51690		5th Intron AltD in dicots (GTT|GTA)
AC166315_29	Ribosomal protein L13e IPR001380:Ribosomal protein L13e	57488176	AltD	*Mt*,*Ot*			
AC136506_20	Peptidylprolyl isomerase, FKBP-type IPR001179:Peptidylprolyl isomerase, FKBP-type IPR001440:TPR repeat	83665229	AltD	*Mt*,*Gm*			
CT868696_4	RNA-binding region RNP-1 (RNA recognition motif) IPR000504:RNA-binding region RNP-1 (RNA recognition motif)	13369035	ExonS	*Mt*,*Ot*			
AC145449_47	IPR001753:Enoyl-CoA hydratase/isomerase Enoyl-CoA hydratase/isomerase::short desc	33106093	ExonS	*Mt*,*Lj*	At3g24360	LOC_Os06g39344	Mutually exclusive exons
CU019604_28	Carboxypeptidase regulatory region IPR008969:Carboxypeptidase regulatory region	6072552	IntronR	*Gm*,*Ot*			
AC175311_20	Peptidase A22B, minor histocompatibility antigen H13 IPR007369:Peptidase A22B, minor histocompatibility antigen H13 IPR006639:Peptidase A22, presenilin signal peptide	7560782	IntronR	*Mt*,*Ot*			

AC166315_30	D111/G-patch; Zinc finger, C2H2-type IPR000467:D111/G-patch IPR007087:Zinc finger, C2H2-type	7147078	IntronR	*Gm*,*Ot*	At5g26610	LOC_Os04g02500	IntronR conserved in Legume, *At*, *Os*
AC151460_3	IPR001471:Pathogenesis-related transcriptional factor and ERF Pathogenesis-related transcriptional factor and ERF::short desc	27405501	IntronR	*Mt*	At1g53910	LOC_Os06g09390, LOC_Os09g26420, LOC_Os03g08470	3-UTR intronR conserved. AP2 domain, ethylene response element binding protein
AC149601_13	Protein kinase IPR000719:Protein kinase IPR002290:Serine/threonine protein kinase IPR001245:Tyrosine protein kinase IPR008271:Serine/threonine protein kinase, active site IPR011009:Protein kinase-like	4292765	IntronR	*Gm*,*Ot*			
AC144728_30	SecA protein IPR011115:SecA DEAD-like IPR011130:SecA preprotein cross-linking region IPR011116:SecA Wing and Scaffold IPR000185:SecA protein	19348856	IntronR	*Gm*,*Ot*			
AC144478_29	phnP protein , related	13370669	IntronR	*Mt*,*Gm*			
AC134242_43	IPR000504:RNA-binding region RNP-1 (RNA recognition motif) RNA-binding region RNP-1 (RNA recognition motif)::short desc	89246673	IntronR	*Mt*	At2g21660, At4g39260	LOC_Os12g43600	Glycine rich protein?
AC152402_23	IPR000504:RNA-binding region RNP-1 (RNA recognition motif) RNA-binding region RNP-1 (RNA recognition motif)::short desc	83665452	IntronR ExonS	*Mt*/*Gm*	At4g27000	LOC_Os04g53440	RBP45 ExonS conserved in *At*; IntronR conserved in *Os*
CR954193_8	Splicing factor, arginine/serine-rich 16 , related	20273599	IntronR/ AltD	*Mt*	At4g36980	LOC_Os03g27840	IntronR conserved in Rice. AltD conserved in *Mt*/*At*/*Os*
AC124951_11 (AC159962_17)	Carbonic anhydrase, prokaryotic and plant IPR001765:Carbonic anhydrase, prokaryotic and plant	7766330; 7766330	IntronR; AltD	*Mt*,*Ot*; *Mt*,*Gm*,*Ot*, *Lj*			Legume specific AS in 3-UTR intron (AC159962_17 only 1 nt diff over 4322 nt)
